# A reliable approach for identifying acute lymphoblastic leukemia in microscopic imaging

**DOI:** 10.3389/frai.2025.1620252

**Published:** 2025-07-17

**Authors:** Mimosette Makem, Levente Tamas, Lucian Bușoniu

**Affiliations:** ^1^Signal, Image, and Systems Laboratory, Department of Medical and Biomedical Engineering, HTTTC EBOLOWA, University of Ebolowa, Ebolowa, Cameroon; ^2^Department of Automation, Technical University of Cluj-Napoca, Cluj-Napoca, Romania

**Keywords:** leukemia classification, image processing, CNN, disease detection, data augmentation

## Abstract

Leukemia is a deadly disease, and the patient’s recovery rate is very dependent on early diagnosis. However, its diagnosis under the microscope is tedious and time-consuming. The advancement of deep convolutional neural networks (CNNs) in image classification has enabled new techniques in automated disease detection systems. These systems serve as valuable support and secondary opinion resources for laboratory technicians and hematologists when diagnosing leukemia through microscopic examination. In this study, we deployed a pre-trained CNN model (MobileNet) that has a small size and low complexity, making it suitable for mobile applications and embedded systems. We used the L1 regularization method and a novel dataset balancing approach, which incorporates HSV color transformation, saturation elimination, Gaussian noise addition, and several established augmentation techniques, to prevent model overfitting. The proposed model attained an accuracy of 95.33% and an F1 score of 0.95 when evaluated on the held-out test set extracted from the C_NMC_2019 public dataset. We also evaluated the proposed model by adding zero-mean Gaussian noise to the test images. The experimental results indicate that the proposed model is both efficient and robust, even when subjected to additional Gaussian noise. The comparison of the proposed MobileNet_M model’s results with those of ALNet and various other existing models on the same dataset underscores its superior efficacy. The code is available for reproducing the experimental results at https://tamaslevente.github.io/ALLM/.

## 1 Introduction

Leukemia is a serious type of blood cancer marked by the uncontrolled and excessive generation of abnormal and immature white blood cells within the bone marrow. According to the Lymphoma and Leukemia Society (LLS, n.d.), between 2013 and 2017, leukemia was the sixth leading cause of cancer deaths in males and the seventh in females in the United States. In 2023, an estimated 13.900 males and 9.810 females may die from leukemia in the US (Blood Cancer CH, 2023). The 2020 report from the World Health Organization estimated 1,342 deaths in Romania alone (Leukemia in Romania, 2020). These statistics show the deadly nature of leukemia. Nevertheless, early diagnosis of this disease is helpful for the recovery of patients, particularly children (Bain et al., 2017). Consequently, early and accurate identification of leukemia is crucial to lowering its death rates.

Usually carried out by laboratory technicians, blood specimen analysis under a microscope is a vital and reasonably priced method among several leukemia diagnosis techniques (Das et al., 2021; Makem and Tiedeu, 2020; Paiva et al., 2018; Walker et al., 1994; Dorfman et al., 2018; Alexander and Mullighan, 2021). However, this procedure requires technicians to perform visual analysis and leucocyte classification, a task that is both labor-intensive and time-consuming. Researchers have developed several computational image analysis techniques to overcome these challenges and diagnose leukemia using blood smear pictures. Traditional computerised image analysis methods for leukemia diagnosis usually consist of preprocessing, segmentation, feature extraction, and classification (Mohapatra and Patra, 2010; Putzu et al., 2014; Rawat et al., 2015; Singhal and Singh, 2014; Rawat et al., 2017; El Houby, 2018; Mohammed and Abdulla, 2021; Bodzas et al., 2020; Mishra et al., 2019a; Mishra et al., 2019b; Abdulla, 2020; Bhattacharjee and Saini, 2016). Consequently, the efficacy of each phase is contingent upon the efficacy of the preceding stage. Deep learning architectures have proven to be more efficient and accurate for disease detection than traditional methods. They learn and extract complex features directly from the images without a previous segmentation step. A solitary deep learning model can execute both feature extraction and classification tasks in various domains (Eralp and Sefer, 2024). One shortcoming of such models is, however, that they require a large amount of data to yield good performance. We can address the lack of extensive datasets by implementing a transfer learning-based methodology (Das and Meher, 2021).

In this study, we applied we made adaptations to the MobileNet architecture (Howard et al., 2017) to identify the presence of acute lymphoblastic leukemia (ALL) in a collection of microscopic blood smear pictures. The Global Average Pooling, dropout layer, batch normalization layer, and dense layer are used to modify the MobileNet architecture to enhance its ability to differentiate between normal and leukemia blood cells. We applied L1 regularization to improve the model’s generalization. Also, a new dataset augmentation process was used, involving Gaussian noise and existing augmentation techniques. The following is a list of this study’s principal contributions:

1. The HSV color space is used, with the saturation removed and additional Gaussian noise;
2. The base MobileNet architecture performs compression to detect ALL;
3. Transfer of classification knowledge learnt on the ImageNet dataset to the acute lymphoblastic leukemia classification task;
4. Development of an efficient and robust model to small Gaussian noise;
5. Evaluation of the classification efficacy of the suggested acute lymphoblastic leukemia detection against contemporary methodologies.

We structure the remainder of the work into five sections. Section 2 presents related works, followed by the proposed technique in Section 3. Section 4 describes experimental validation and discussion. Section 5.2 delineates conclusions and future work.

## 2 Related work

### 2.1 Traditional methods

Over the years, researchers have developed traditional methods for diagnosing lymphoblastic leukemia. The authors focused mainly on the classification of normal or healthy leukocytes from abnormal or lymphoblast leukocytes. Mohapatra and Patra (2010) suggested a framework for the identification of acute leukemia. This approach begins by applying a selective filter to the blood smear picture and transform the resultant image into the L*a*b color space (Putzu et al., 2014). The K-means algorithm is subsequently employed on the transformed image to isolate the white blood cell nucleus from the other elements. Each nucleus of a white blood cell was segmented into a sub-image. Subsequently, features related to forms and textures was retrieved from the nucleus. The SVM classifier categorizes nucleus pictures as healthy or leukemic based on the retrieved attributes. The algorithm was evaluated by considering 108 blood smear images collected at Ispat General Hospital in Rourkela, Odisha, and at the University of Virginia. The authors reported a lymphoblast detection accuracy of 95%.

Putzu et al. (2014) also created a method for identifying and categorizing white blood cells in pictures of blood smears. The original RGB image is transformed into grayscale and CMYK color spaces, followed by the application of enhancement techniques, including histogram equalization and linear contrast stretching, to increase the image quality. The Zack algorithm is employed for thresholding to segment white blood cells. The morphological opening operator was employed to eliminate the residual undesirable objects. Ultimately, 30 morphological variables, four chromatic features, and 16 textural features were retrieved from the nucleus and cytoplasm regions to categorize cells as normal or aberrant via an SVM classifier and cross-validation. Putzu et al. (2014) suggested an approach that achieved an accuracy of 93% for 33 images from the ALL-IDB1 database (Mondal et al., 2021) acquired under the same conditions. The main limitation of this approach is that images acquired with different cameras and different lighting conditions are not considered.

Rawat et al. (2015) suggested an approach to differentiate lymphoblastic cells from healthy lymphocytes. Their method involved initially segmenting the leukocytes from other blood cells, followed by the separation of the isolated leukocytes into their nucleus and cytoplasm components. Subsequently, distinct texture characteristics from the grayscale co-occurrence matrix and shape features are retrieved from the nucleus and cytoplasm areas, respectively. The collected features were identified using a binary Support Vector Machine (SVM) to identify the presence of lymphoblast cells (leukemic cells). The method of Rawat et al. (2015) was tested by considering 196 images of the ALL-IDB2 database and a classification accuracy of 89.8% was obtained. That method is therefore limited in terms of detection accuracy.

Singhal and Singh (2014) conducted a comparative investigation of the performance of two types of texture feature descriptors. In their study, the original RGB image is initially transformed into the HSI color space, followed by the extraction of the white blood cell nucleus with the use of a manual threshold on the S component. Two categories of texture feature descriptors, specifically the local binary pattern (LBP) and the grayscale co-occurrence matrix are used for feature extraction, followed by the polynomial kernel SVM classifier for classification. The ALL-IDB2 images database was considered to evaluate the performance of this algorithm. The classification accuracy of the features that were extracted using the LBP was 93.84%. A classification accuracy of 87.30% was attained utilizing the GLCM features. They evaluated the performance of this algorithm using the ALL-IDB2 images database. Although giving an accuracy of detection in the order of 93%, this classification system is unsuitable for computer-aided diagnosis of leukemia due to the manual determination of the threshold for leukocyte nucleus extraction.

Rawat et al. (2017) proposed a hybrid hierarchical diagnostic support system that analyzes the blood smear image for rapid detection of acute lymphoblastic leukemia. This system not only distinguishes between healthy leukocyte cells and leukemia cells, but also categorizes ALL cells into subtypes. The leukocyte nucleus and cytoplasmic components are extracted from the original smear image in this system. The properties of texture, color, and form of the segmented nucleus and cytoplasm are retrieved for the classification of lymphoblasts. A PCA-based dimensionality reduction module is employed to decrease the size of the retrieved features. The classifiers SVM, KNN, PNN, ANFIS, and SSVM are employed in a hierarchical sequence for classification purposes. The computer aided diagnosis system of Rawat et al. (2017) evaluated with 260 images of ALL-IDB was able to classify cancerous leukocytes from healthy leukocytes while classifying cancerous leukocytes into subtypes according to the FAB classification with an average accuracy of 97.6%. This algorithm is powerful, but extremely slow to execute due to the number of components that need to be executed.

El Houby (2018) focused their interest on identifying healthy leukocytes from lymphoblast leukocytes. In their method, shape, texture, and color attributes were collected from nuclear regions, cytoplasm, and whole leukocytes respectively, previously segmented from other cells present in blood smear images and cropped into sub-images. Ant colony optimization (ACO) was employed to choose characteristics derived from the segmented cellular components to enhance classification performance. Classification was performed using decision tree (DT), K-nearest neighbor (K-NN), Naïve Bayes (NB), and support vector machine (SVM) algorithms. Their proposed classification system evaluated with 260 images of the ALL-IDB2 database showed classification accuracy of 96.25%, achieved with the DT classifier. The main shortcoming of this classification system is that it was tested with a small number of images.

Mohammed and Abdulla (2021) focused on the development of an automatic system to assist in the identification of ALL cells. The suggested methodology comprises two phases. The first phase emphasizes the segmentation of leukocytes. The second stage recovers characteristics such as shape, geometry, statistics, and discrete cosine transform from the segmented cells. They apply KNN, SVM, and NB to the retrieved characteristics to classify the segmented cells as either normal or pathological. This method’s efficacy was assessed using the ALL-IDB2 blood smear image database. The experimental results achieved a maximum accuracy of 97.45% with the SVM. Although performing well, this algorithm should be evaluated by considering a different image set for validation.

Bodzas et al. (2020) created an algorithm for leukemia identification, employing a three-tiered filtering procedure for the segmentation of the nucleus and cytoplasm of white blood cells. Additionally, the algorithm considered the detection and separation of agglomerated white blood cells. Sixteen shape and texture parameters were retrieved from the segmented regions to enable the classifier to differentiate between normal and diseased cells. The employed classifier was Support Vector Machine (SVM). The University Hospital in Ostrava’s Department of Haemato-Oncology supplied a private image collection for training and testing the system created by Bodzas et al. (2020). The image set consisted of 33 images, 18 of which were acquired from healthy people and 13 from people with ALL. According to the authors, their system achieved a classification accuracy of 96.72%. This algorithm requires an expansion of the database used for testing and training.

Looking at the traditional computer-vision methods presented so far, that they are mainly focused on segmentation and the extraction of specific features. However, nucleus and cytoplasm identification and extraction are a challenging task due to the contrast variations of different types of leucocyte, which leads to a low accuracy of the expected result. Also, it not trivial to extract a suitable feature set able to distinguish health leucocytes from cancer leucocytes.

### 2.2 Deep learning methods

Recent proposals have emerged for the detection and categorization of ALL using deep learning approaches (Shah et al., 2021; Deshpande et al., 2020; Loey et al., 2020) due to the advancements in artificial intelligence and extensive data analysis. Vogado et al. (2018) used pretrained CNN models (AlexNet, VGG_f, and CaffeNet) for feature extraction and employed SVM for classification in an ALL detection system. The hybrid dataset, which included ALL-IDB, CellaVision, and leukocyte mixed databases, yielded an average accuracy of 99.20%. In order to identify white blood cells in blood smear images and categorize them as either leukemia or healthy, Di Ruberto et al. (2020) presented an algorithm. The white blood cell detection step employed the H and S color components, Otsu’s thresholding method, a new object detection technique, and the watershed method. The AlexNet convolutional network was used to extract 3 different feature vectors of white blood cells previously segmented and cropped into a sub-image. The retrieved feature vectors are classified using three distinct linear SVMs, and their results are amalgamated through a voting process. The classification system was tested on 33 images of the ALL-IDB2 database and an accuracy of 94.1% was obtained. This classification system is limited because it was built and assessed based on smear images captured with the identical camera and under uniform illumination circumstances.

Shafique and Tehsin (2018) employed the pre-trained deep convolutional neural network AlexNet to identify LLA and categorize it into its subtypes (L1, L2, and L3). This algorithm involved modifying the architecture of the AlexNet network by substituting the final three layers of the pre-trained model with a new fully connected layer containing 1,024 neurones, succeeded by a ReLU layer and an additional fully connected layer, wherein all units were interconnected to the output probabilities of two classes via the *softmax* function. For the classification of the LLA into subtypes, the last fully connected layer with 2 output probability classes was changed to 4 output probability classes. The ALL-IDB2 database, comprising 260 smear images, was utilized for the assessment of their algorithm. The outcomes attained an average categorization accuracy of 96.06%. In their study, the removal of noise in the original image was omitted, yet the presence of noise results in erroneous features and therefore impacts on the performance of the classification system. On the other hand, a limited number of images was considered for training and testing, yet this has a negative effect on the learning of the deep network.

Claro et al. (2020) developed two deep convolutional neural networks (DCNs) designated Alert Net-R and Alert Net-X to diagnose myeloid and acute lymphoblastic leukemia. The Alert Net-R network was designed by inserting residual structures similar to those of the ResNet into the original Alert Net architecture. The Alert Net-X network was built using the technology implemented in *Xception* by Nvidia. In addition, a data augmentation procedure was employed to enhance the volume of the training dataset. The designed RNC has been trained and tested with 16 image databases including 2,415 images, and an overall accuracy of classification equal to 97.23% has been obtained. This accuracy is good, considering that it is obtained by considering a heterogeneous image database. However, its implementation requires high resources.

Hegde et al. (2019) assessed the impact of characteristics derived from conventional image processing and the pre-trained CNN AlexNet on neural network classifier. In their study, the neural network classifier was used to classify abnormal and normal WBC, also to classify normal WBC in their sub-types. Eralp and Sefer (2024), however, suggested a hybrid transfer learning approach in which ResNet18 and MobilenetV2 are hybridized based on their proposed weight factor. The ALL-IDB1 and ALL-IDB2 was considered to evaluate the performance of the methods. According to the authors, when the dataset is divided into 50% training and 50% testing, the computer-aided system’s performance is limited.

Nabilah et al. (2020) proposed a comparison analysis of three pre-trained convolutional neural networks, specifically VGG, GoogleNet, and AlexNet, for the identification of acute lymphoblastic leukemia. That study showcased VGG as the best architecture based on its testing and training accuracy. The main limitation is that only a few image samples were considered in this study. In contrast, Mondal et al. (2021) developed a weighted ensemble classifier for ALL detection by applying transfer learning to pre-trained CNNs including VGG-16, Xception, MobileNet, InceptionResNet-V2, and DenseNet-121. The dataset C-NMC-2019 was utilized to train and evaluate the constructed model, yielding an average accuracy of 86.2%.

Ullah et al. (2021) adopted the efficient channel attention module to enhance the VGG-16 architecture’s ALL detection. The developed model was assessed using the C-NMC-2019 dataset and attained an accuracy of 91.1%, indicating a need for enhancement. Jawahar et al. (2022) introduced a CNN model called ALNett, which is founded on a depth-wise convolutional architecture. On the training folder of the C-NMC-2019 dataset, the ALNett model achieved an F1_score of 0.96 and a classification accuracy of 91.31%. Magpantay et al. (2022) developed a transfer learning approach utilizing Yolov3 for the classification of ALL cells and normal cells. Only 300 images selected from the C-NMC-2019 dataset were considered in this study, and the model achieved a training accuracy value of 97.2% and an mAP value of 99.8% on testing images. This method considered few images of a large dataset for the training and testing the models. However, an effective and resilient leukemia diagnosis system must provide results for a substantial volume of smear images, including those from alternative leukocyte datasets.

Yolov5 was applied for detection and count of blood cell in Rohaziat et al. (2022). Priyanka et al. (Liu and Hu, 2022) proposed a model named LeuFeatx, an adapted, fine-tuned feature extractor model based on VGG16. LeuFeatx demonstrated promising performance both in the leukemia subgroup classification and the binary classification. The ALL-IDB2 dataset was utilized for binary classification, yielding an accuracy of 96.15%. Abhishek et al. (2025) proposed the fuzzification of pretrained convolutional neural networks with the Gompertz function; the developed methodology categorized blood smear pictures into five classifications: AML, CML, ALL, CALL, and normal. Similarly, VGG16 and XceptionNet models were combined for classification of four type of diabetic eye disease (Hasan et al., 2025). Election-Base Chameleon Swarn algorithm was used on multiscale adaptive and attention-base DCNN method for leukemia detection (Gokulkannan et al., 2024).

Existing methods for leukemia diagnosis based on transfer learning have not considered the complexity of the pre-trained CNN, including the parameter count. This leads to a resource-intensive model that is unsuitable for embedded systems and computers with limited performance. To address this limitation our proposed models are based on MobileNet (Howard et al., 2017), which has a smaller size and complexity models, and is therefore suitable for mobile applications and embedded systems. Conversely, MobileNet has superior classification accuracy compared to alternative lightweight approaches.

## 3 Proposed methodology

**Figure 1:**
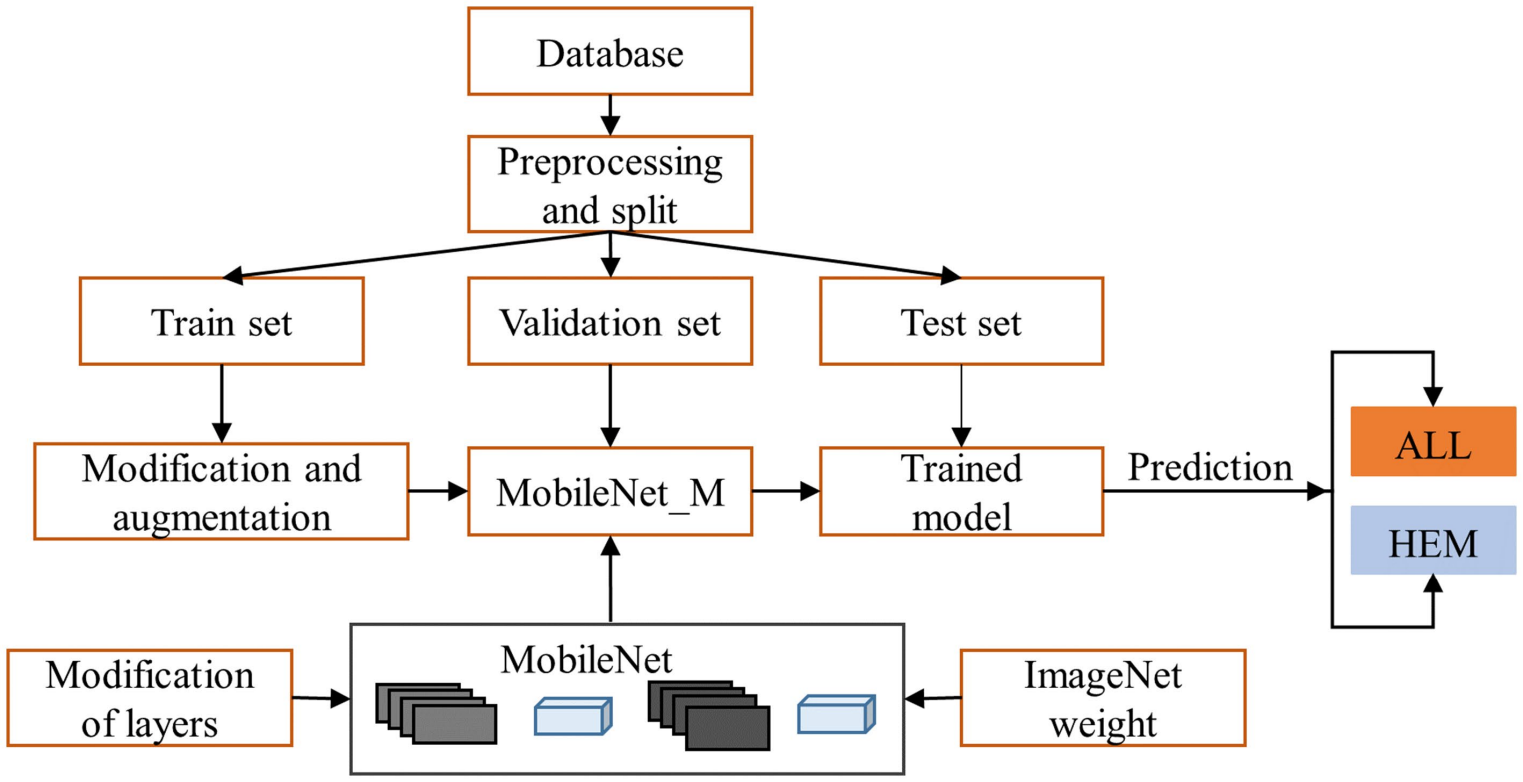
Proposed methodology of leukemia diagnosis.

The overarching schematic of the suggested methodology is illustrated in Figure 1. The proposed methodology has two stages. The first stage includes dataset processing, modification, and augmentation. The second stage performs transfer learning classification using MobileNet. The sequel will provide explanations for all the components in the figure.

### 3.1 Dataset description

The pictures used in this paper are from the public ISBI 2019 database available on Kaggle (Gupta and Gupta, 2019). The ISBI 2019 database contains blood smears with resolution of 450 × 450 × 3 pixels, designed to distinguish leukemic B lymphoblast cells (ALL) from normal B lymphoid precursors (HEM). The training, preliminary, and final test sets make up this database. The training set comprises 10,661 white blood cell images, categorized into 7,272 leukemic images (ALL) and 3,389 healthy images (Hem). These images are from 47 leukemia patients and 26 healthy patients. We divide the training set into three separate folders. The preliminary test set contains 1,219 ALL and 648 HEM images extracted from the blood smears of 13 leukemia patients and 15 healthy patients, respectively. The final test set included 2,586 white blood cell pictures from nine patients with acute lymphoblastic leukemia and eight healthy individuals.

**Figure 2:**
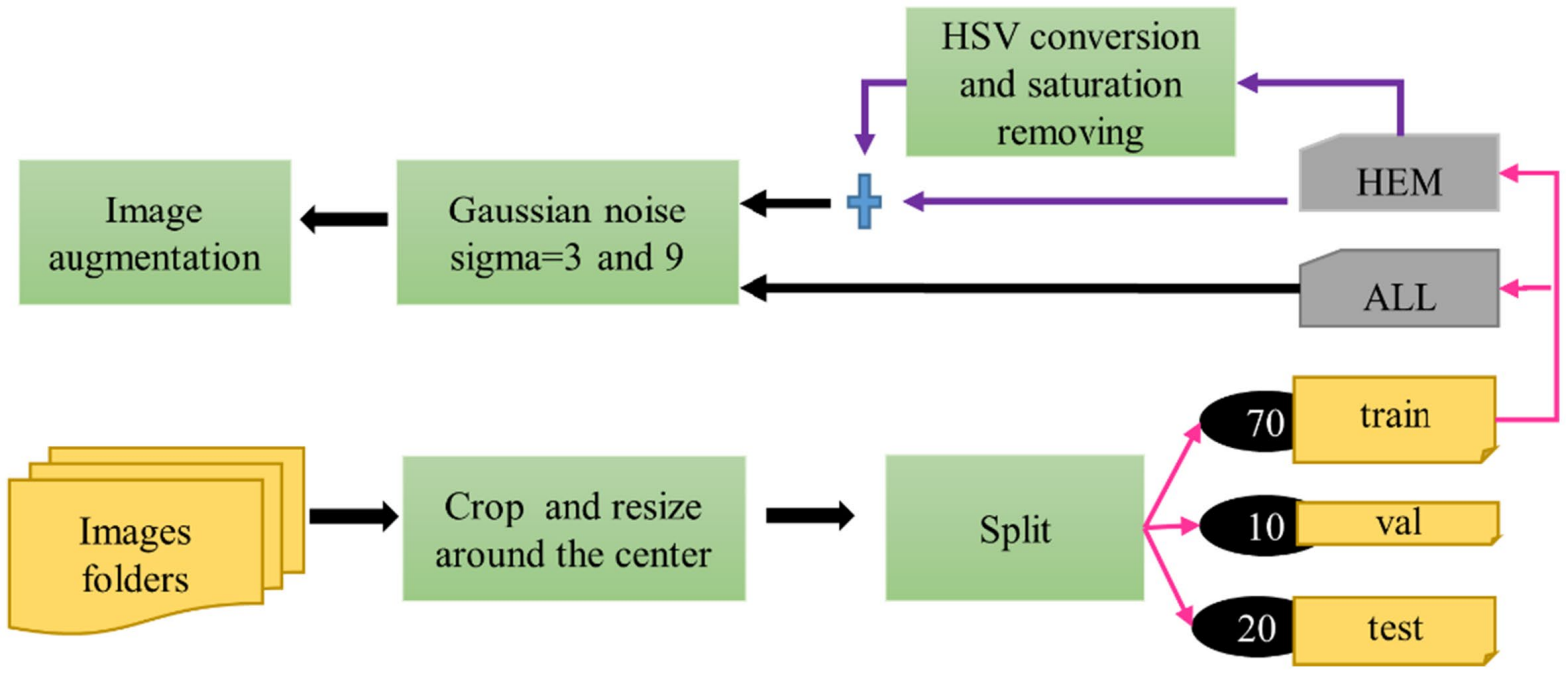
Preprocessing steps of the proposed approach.

Figure 2 illustrates the preprocessing steps and modifications applied to the database, which we discuss next:

### 3.2 Modification of dataset

In the study, the three separate folds of the ISBI 2019 database training set, which are individually stratified into HEM and ALL folds, were independently preprocessed and divided using percentages [70, 10, 20%] into training, then validation and test sets. Table 1 displays the distribution of each fold. All images in the dataset underwent cropping and scaling adjustments. We executed the cropping from the center of the original image to preserve the entire white blood cell. We resized the resulting image to 224*224*3 to meet MobileNet’s input requirements. Table 1 reveals an imbalanced class problem in the ISBI 2019 database. Indeed, the ratio of the total number of ALL pictures to the number of HEM pictures is consistently 2 across all folders in the training set. According to Mayouf et al. (2020), a dataset is qualified as slightly unbalanced when this ratio is in the range [1.5, 3]. Class imbalance in a database affects the model generalization in favor of the majority class (Johnson and Khoshgoftaar, 2019; Kaope and Pristyanto, 2023). There are two ways to rebalance data sets, namely (Orriols-Puig and Bernadó-Mansilla, 2009): one class is oversampled, while the majority class is undersampled. This study took into account the oversampling technique to lessen the disparity between the ALL and HEM classes in the training set for the three folders. This procedure was selected because, unlike the undersampling strategy, it does not diminish the size of the data collection. The oversampling process was based on converting each image of the HEM class of the train set into the HSV color space; then the saturation was removed from the resulting image. These two operations doubled the number of images in the HEM class, as shown in Table 2, without any duplication of information.

**Table 1:** Train, test, and validation distribution over the different batches denoted as folds.

Folds	Train	Val	Test	Total
Fold 0	ALL: 1677 HEM: 791	ALL: 239 HEM: 113	ALL: 481 HEM: 226	ALL: 2397 HEM: 1130
Fold 1	ALL: 1692 HEM: 814	ALL: 241 HEM: 116	ALL: 485 HEM: 233	ALL: 2418 HEM: 1163
Fold 2	ALL: 1719 HEM: 767	ALL: 245 HEM: 109	ALL: 493 HEM: 220	ALL: 2457 HEM: 1096

**Table 2:** Images in the HEM class before and after class balance.

Folds	Number of HEM images in train set	
	Before balance process	After balance process
Fold 0	791	1,582
Fold 1	814	1,628
Fold 2	767	1,534

### 3.3 Data augmentation

**Figure 3:**
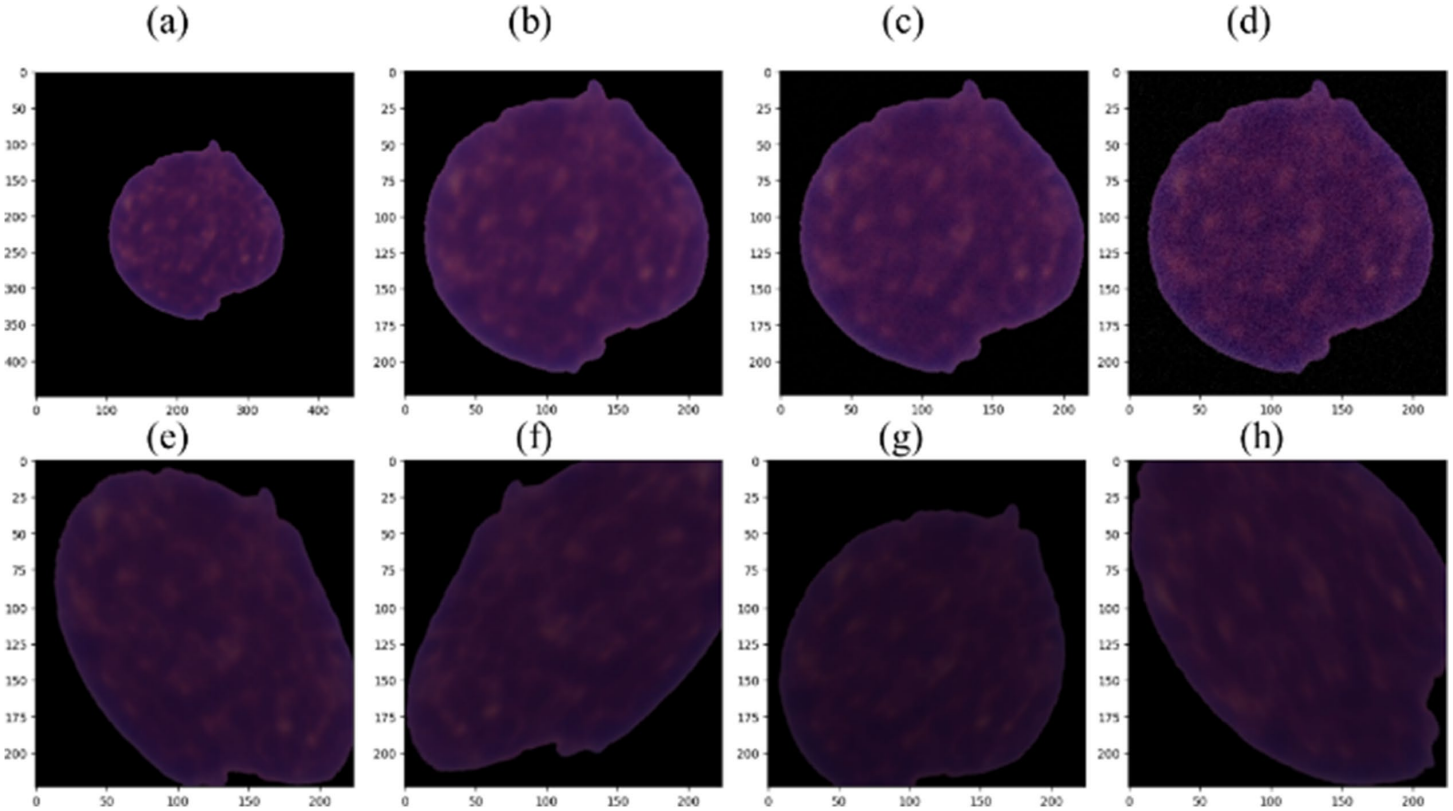
**(a)** Example of ALL image; **(b)** cropped and resized ALL image; **(c)** image with Gaussian noise using sigma = 3; **(d)** image with Gaussian noise using sigma = 9; **(e–h)** augmented images.

**Figure 4:**
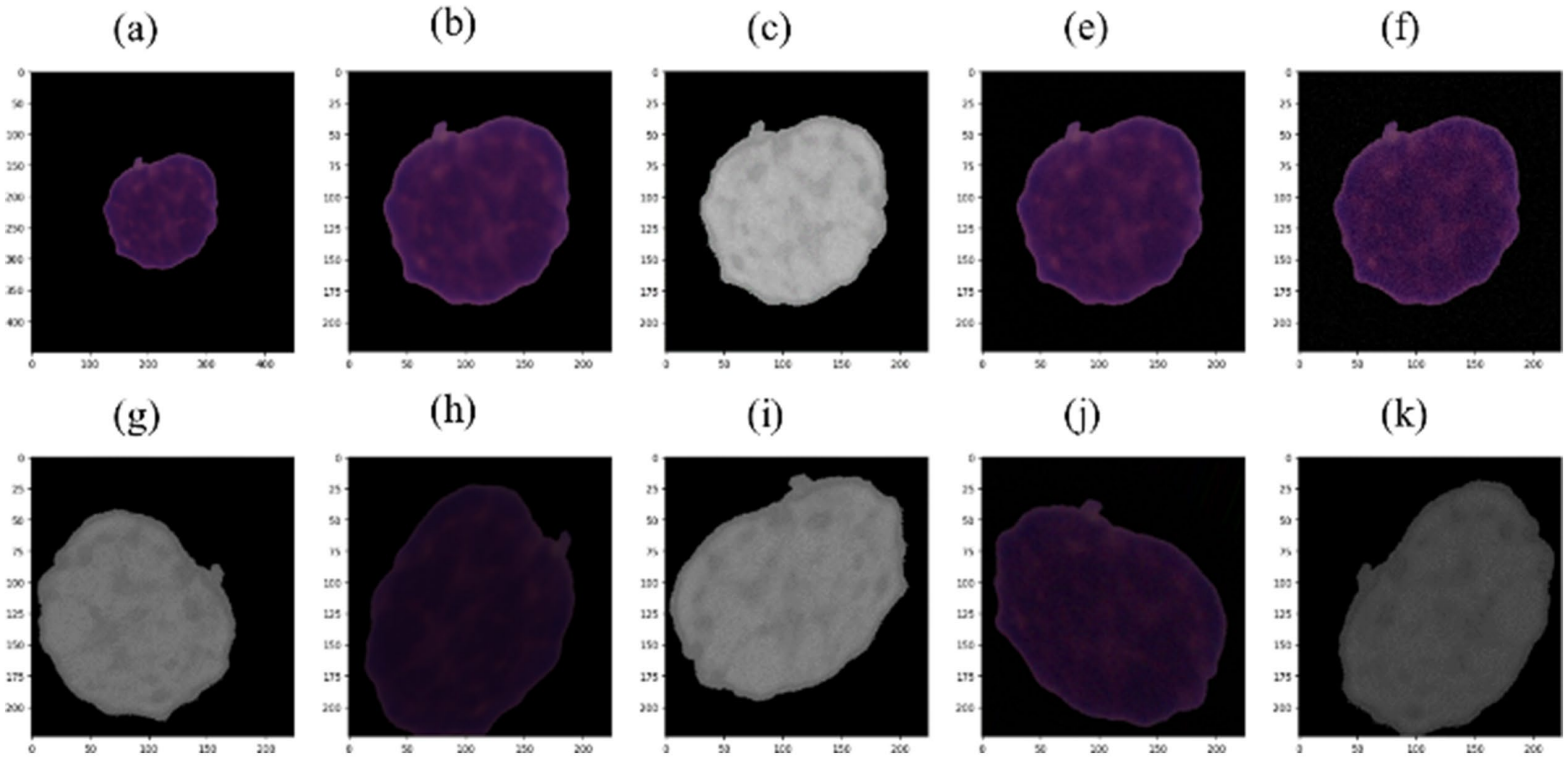
**(a)** Example of HEM picture; **(b)** cropped and resized picture; **(c)** HSV picture with saturation removed; **(e)** picture with Gaussian noise using sigma = 3; **(f)** picture with Gaussian noise using sigma = 9; **(g–k)** augmented pictures.

Prior research has demonstrated the beneficial effects of image augmentation on enhancing the characteristics and diversity of a dataset. We therefore apply the following augmentation procedures to our data. Gaussian noise with a mean of zero and standard deviations of 3 and 9 was injected in equal proportions in the training dataset for augmentation. This step aimed to build a model that is insensitive to Gaussian noise. The sigma values were selected experimentally. Other augmentation steps, such as rotation of 45 degrees, horizontal flip, brightness range [0.4, 0.8], width shift range (0.1), height shift range (0.1), zoom range [0.8, 1], shear range (3), and rescale (1/255), were applied to generate new images during the training phase. Pixel values of all the images of our dataset were scaled to the range [0, 1]. Figures 3, 4 illustrate some image samples of the ISBI 2019 database and the augmentation.

### 3.4 MobileNet

The transfer learning technique is based on choosing a pre-trained model and fine-tuning it to solve a new classification problem. The main benefits of transfer learning are sharing knowledge and saving training time and resources (Sarkar et al., 2018). Due to its foundation in depthwise separable convolution, MobileNet was selected as the pre-trained convolutional neural network for classification, so it is extremely efficient and has a low computational cost compared with other standard convolution-based models.

**Figure 5:**
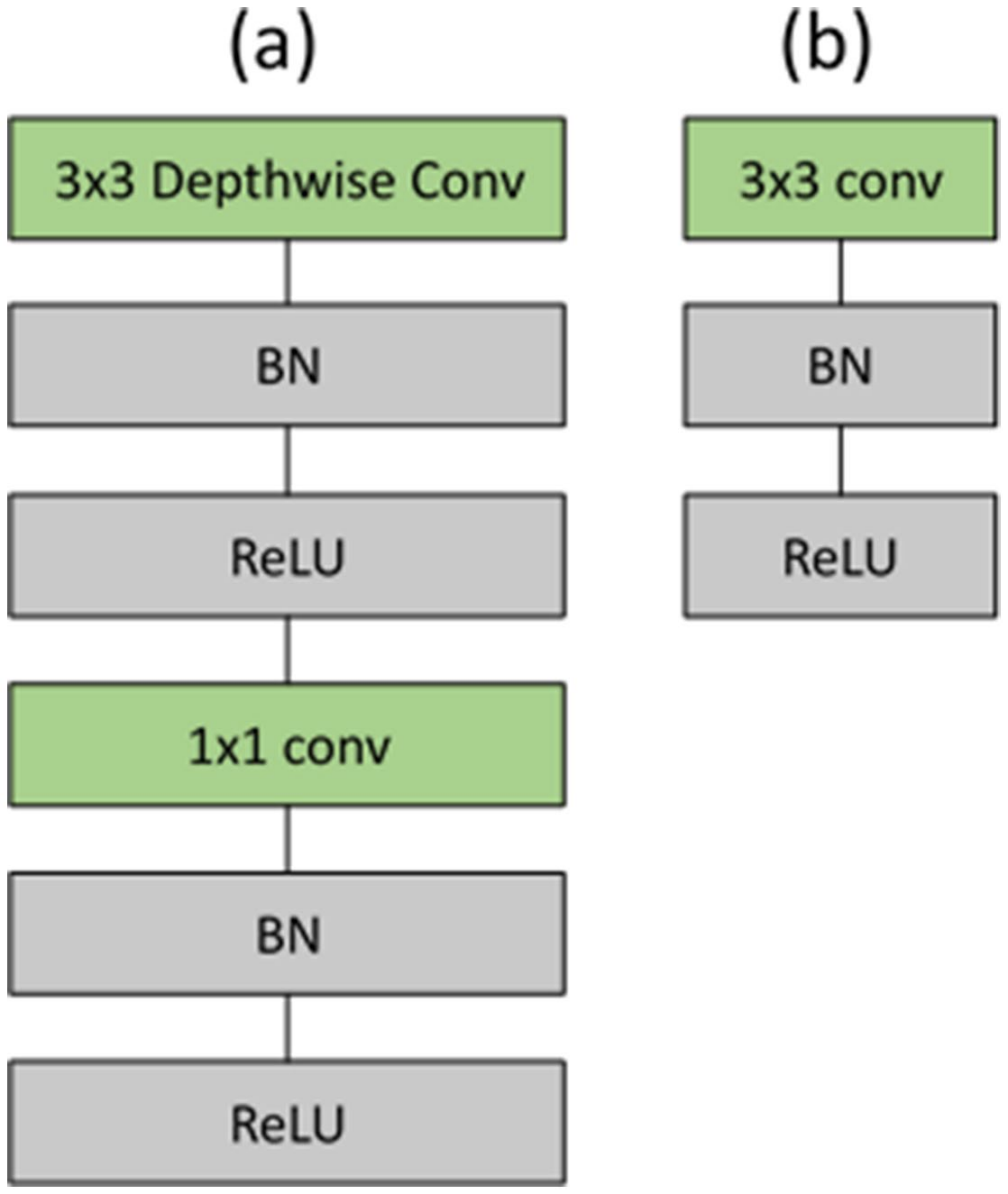
**(a)** Depthwise separable convolutions consisting of depthwise and pointwise layers, succeeded by batch normalization and ReLU; **(b)** standard convolutional layer accompanied by batch normalization and ReLU.

MobileNet comprises convolutions, depthwise separable convolutions, batch normalization, ReLU activations, and fully connected layers (Ashwinkumar et al., 2021). Among these layers, depthwise separable convolution serves as the fundamental layer of the MobileNet model, minimizing both the number of parameters and computing expense by decomposing a normal convolution into a depthwise convolution. In Table 3, the initial MobileNet design is displayed. The batch normalization and ReLU activations follow each layer of the architecture, as illustrated in Figure 5. The MobileNet depthwise separable convolutional layer is broken into 3×3 depthwise convolution filters and 1×1 pointwise convolution. A single 3×3 depthwise convolution filter is applied to each image channel, followed by a 1×1 pointwise convolution to generate a linear combination of the output as per (Howard et al., 2017). The Mathematical expression of depthwise convolution with one filter per input channel can be defined as shown Equation 1:


(1) $$\hat{G}=\sum_{i,j}{\hat{K}}_{i,j,m}F_{k+i-1,l+j-1,m}$$

where *k* and *l* are locations in the $m_{\mathrm{th}}$ feature map, $\hat{K}$ represents the depthwise convolutional kernel of size $D_{k}\times D_{k}\times M$ , $\hat{G}$ is the output feature map obtained when the $m_{\mathrm{th}}$ filter in $\hat{K}$ is applied to the $m_{\mathrm{th}}$ channel in F .

**Table 3:** Architecture of MobileNet.

Type/Stride	Filter Shape	Input Size
Conv/s2	3 ×3×3×32	224 ×224×3
Conv dw/s1	3 ×3×32 dw	112 ×112×32
Conv/s1	1 ×1×32×64	112 ×112×32
Conv dw/s2	3 ×3×64 dw	112 ×112×64
Conv /s1	1 ×1×32×128	56 ×56×128
Conv dw/s1	3 ×3×128 dw	56 ×56×128
Conv /s1	1 ×1×32×128	56 ×56×128
Conv dw/s2	3 ×3×128 dw	56 ×56×128
Conv/s1	1 ×1×32×256	28 ×28×128
Conv dw/s1	3 ×3×256 dw	28 ×28×256
Conv/s1	1 ×1×32×256	28 ×28×256
Conv dw/s2	3 ×3×256 dw	28 ×28×256
Conv /s1	1 ×1×32×512	14 ×14×256
Conv dw/s1 Conv/s1	3 ×3×512 dw 1 ×1×512×512	14 ×14×512 14 ×14×512
Conv dw/s2	3 ×3×512 dw	14 ×14×512
Conv/s1	1 ×1×512×1024	7 ×7×512
Conv dw/s2	3 ×3×1024 dw	7 ×7×1024
Conv/s1	1 ×1×1024×1024	7 ×7×1024
Avrg pool/s1	Pool 7 ×7	1 ×1×1000
FC/s1	1 1024×1000	1 ×1×1024
Softmax/s1	Classifier	1 ×1×1000

Total parameters: 4,253,864. Trainable parameters: 4,231,976. Non-trainable parameters: 21,888. size≈ 16 MB.

### 3.5 MobileNet_M: extension of MobileNet

In the proposed work, we replaced the MobileNet average pooling, fully connected, and softmax layers with a set of layers appropriate for our classification problem. The obtained model was called MobileNet_M. Table 4 illustrates the structure of layers used to solve our classification problem. In this structure, the dropout layer is used to randomly deactivate a fraction of the neurons to avoid overfitting, and the Batch Normalization (BN) is employed to stabilize the distribution of the dataset during training, hence expediting model. Mathematically, we can define the BN for convolutional neural networks using Equation 2 (Bjorck et al., 2018):


(2) $$O_{b,c,i,j}=\frac{I_{b,c,i,j-\mu_{c}}}{\sqrt{\sigma_{c}^{2}+\varepsilon }}+\beta_{c}\forall b,c,i,j\;\text{and}\;\mu_{c}=\frac{1}{|B|}\sum_{b,i,j}^{\sum }I_{b,c,i,j}$$

where *i* and *j* are the spatial location of the c channel feature map $O_{b,c,i,j}$ and $I_{b,c,i,j}$ are the BN’s output and input, respectively. Notation $\mu_{c}$ represent the means activation of all images in the batch b and $\sigma_{c}$ the standard deviation. *B* encompasses all activations within channel *c* across every feature *b* in the complete mini-batch and all spatial locations *i,j*. Finally, $\gamma_{c}$ and $\beta_{c}$ are parameters for channel-wise affine transformation.

**Table 4:** The suggested MobileNet_M’s architecture.

Layer (type)	Output shape	Parameters
MobileNet	(None, 7, 7,1,024)	3,228,864
GlobalAveragePooling	(None, 1,024)	0
Flatten	(None, 1,024)	0
Dropout	(None, 1,024)	0
Batch normalization	(None, 1,024)	4,096
Dense	(None, 2)	2050

Total parameters: 3,235,010. Trainable parameters: 3,211,074. Non-trainable parameters: 23,936. GPU Memory Requirement: 6.2 MB. Size = 12.3406 MB.

The dense output layer contains two neurons and a SoftMax activation function that gives an output probability for each neuron. The SoftMax function uses a logistic transformation to map the vector of raw outputs from the neural network (z-scores) into probabilities p∈ [0, 1] as defined in Equation 3:


(3) $$\text{softmax}(z)=\frac{\exp(z_{i})}{\sum_{j=1}^{2}\exp(z_{j})}$$

### 3.6 Tunable hyper-parameters of MobileNet_M

This project involved training MobileNet_M using a substantial dataset of images. We initialized all MobileNet layers for transfer learning using the pre-trained MobileNet model from the ImageNet dataset. We initialized the last dense layer with two units using a uniform distribution. We added L1 weight regularization to the dense layer to enhance the model’s generalization, using a regularization parameter of 0.015. During the training phase, we set the starting learning rate at 0.001, and applied a reduction if we observed no improvement in validation loss over 5 epochs. We set the reduction factor to 0.1 and set the minimum learning to 0.000001. We implemented early halting when the model’s validation loss stopped decreasing after 20 epochs, indicating that the model has stopped learning meaningfully. All hyperparameters utilized throughout the training phase are presented in Table 5.

**Table 5:** Training hyper parameters of the proposed model.

Parameters	Values
Epochs	100
Patience of early stopping	20
Patience of learning rate control	5
Optimizer	Adam
Batch size	16
Loss	Categorical cross entropy
Learning rate	0.001 to 0.000001
L1 regularization	0.015

### 3.7 Evaluation metrics

We assessed the efficacy of the suggested model using criteria such as the confusion matrix, accuracy, precision, recall, F1 score, and AUC. The confusion matrix has the advantage of quickly determining the effectiveness of a classification system. The classification improves as the confusion matrix approaches a diagonal matrix. Accuracy is the proportion of correctly diagnosed leukemia cells (true positives) and healthy cells (true negatives) relative to the total number of cells. Recall is the proportion of true positives identified as opposed to those overlooked. The AUC is the two-dimensional area beneath the complete receiver operating characteristic (ROC) curve. Equations 4–7 provide the mathematical definitions for accuracy, precision, recall, and F1 score:


(4) $$\text{Accuracy}=\frac{\mathrm{TP}+\mathrm{TN}}{\mathrm{TP}+\mathrm{TN}+\mathrm{FP}+\mathrm{FN}}$$

(5) $$\text{Recall}=\frac{\mathrm{TP}}{\mathrm{TP}+\mathrm{FN}}$$

(6) $$\text{Precision}=\frac{\mathrm{TP}}{\mathrm{TP}+\mathrm{FP}}$$

(7) F1_score=2×Precision×RecallPrecision+Recall

TP, TN, FP, and FN denote true positives, true negatives, false positives, and false negatives, respectively. True positives (TP) refer to instances where the model accurately identifies leukemia. False positives occur when the model erroneously predicts the HEM class as the ALL class. False negatives (FN) occur when the model erroneously predicts the ALL class as the HEM class.

## 4 Experimental validation and discussion

### 4.1 Experimental setup

A server with two Intel Xeon Gold 6226R CPUs, four Nvidia A100 40GB GPUs, 756 GB of RAM and Ubuntu 20.04 from which a single card was effectively used for training was used to implement the proposed work. Python 3.9.16 was used with the tensorflow_gpu 2.4.1, keras 2.10, scikit_learn 1.2.2, numpy 1.23.4, and matplotlib 3.7.1 packages. Imgeio 2.30 and imgaug 0.4.0 libraries were employed for modification and Gaussian noise addition to the dataset. Gaussian noise with a mean of zero and sigma values of 2, 5, 6, and 10 was introduced to the test sample where the noise value is different per pixel and per channel (a different value for the red, green and blue channels of the same pixel). The aim was to evaluate our model’s sensitivity to Gaussian noise.

### 4.2 Performance analysis: training step

**Figure 6:**
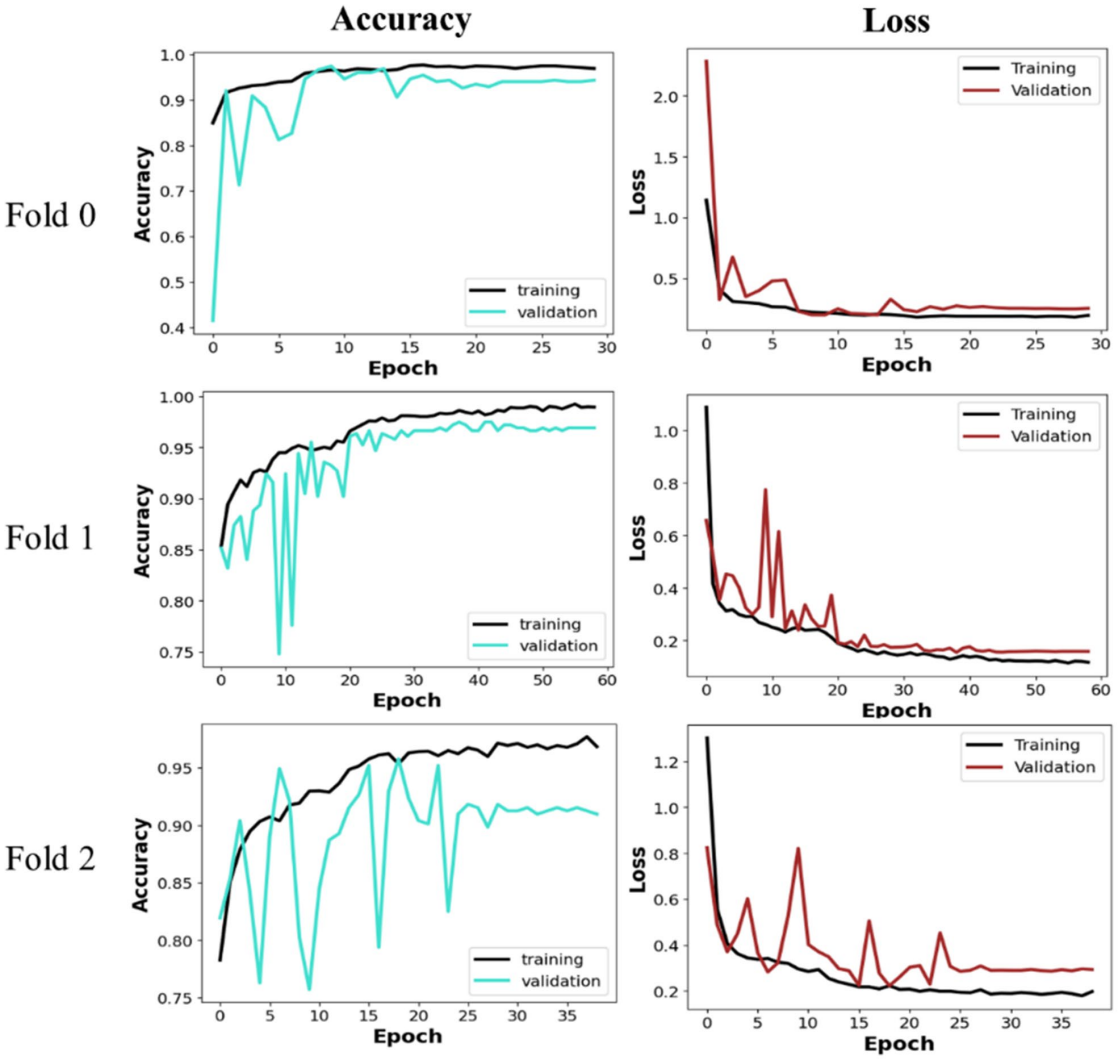
Training and validation curves.

The proposed MobileNet_M model was trained and tested using the three folders of data to assert its efficiency in leukemia detection. As presented in the previous section, MobileNet_M is based on depthwise separable convolution; hence, the L1 weight regulation method was applied to its last dense layer to avoid overfitting. The model had 3,211,074 trainable parameters, which were trained with early stopping and validation loss as the monitoring parameter. Figure 6 depicts the loss and validation accuracy curves for each Fold of the training and validation sets. The graphic indicates that the validation loss begins at a high level and ultimately converges with the training loss across all Folds. We observe a similar trend in the 16th, 23th, and 27th epochs for Folds 0, 1, and 2, respectively. The training and validation accuracy starts with a low value, then increases progressively, and after a few epochs, no further significant improvement is observed. Also, in Figure 6, we can see that the proposed model converges fast: early stopping happens after 30 training epochs for Fold 0, after 60 epochs for Fold 1, and close to 40 epochs for Fold 2. Fold 0 achieves the best and fastest convergence. The average training accuracy was, respectively, equal to 95.83% for Fold 0, 96.60% for Fold 1, and 94.24% for Fold 2.

### 4.3 Performance analysis: prediction phase

**Figure 7:**
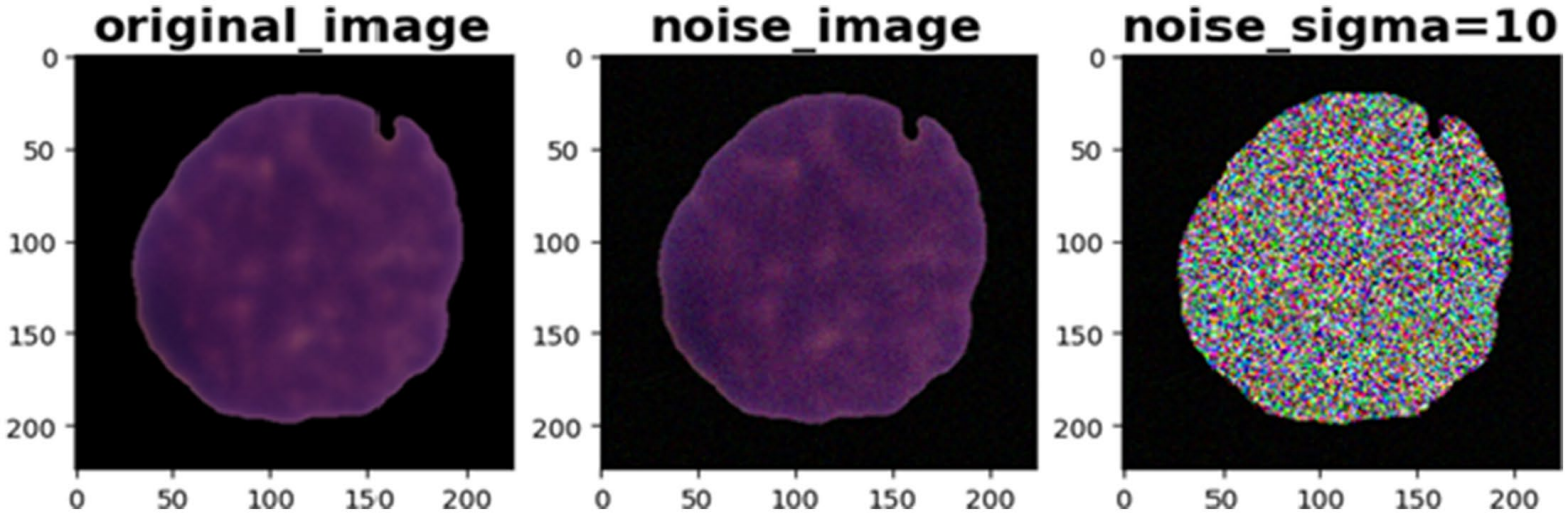
Gaussian noise with sigma = 10 on test sample image.

We introduced Gaussian noise to the test sample, setting the sigma values at 2, 5, 6, and 10. The aim was to evaluate our model’s sensitivity to Gaussian noise. Figure 7 shows a sample test image with Gaussian noise. We computed the evaluation metrics for both noisy and clean datasets. Tables 6–8 present the average obtained result. From Table 6, we notice that for Fold 0, the proposed MobileNet_M models achieved the same accuracy value of 96% on both the clean and noisy test images for sigma values of 2 and 8. All these results illustrate the efficacy and resilience of the proposed model in accommodating fluctuations in the dataset (Folds) and additive Gaussian noise. The prediction time per image was 4.92 ms on a personal computer and 44.68 ms in the remote runtime Google Colab.

**Table 6:** Test classification performance of noisy and clean images in Fold 0.

Test data	Precision	Recall	F1_score	Accuracy	AUC
**Sigma = 0**	**0,96**	**0,95**	**0,96**	**0,96**	**0,954**
Sigma = 2	0,95	0,95	0,95	0,96	0,952
Sigma = 5	**0,94**	0,95	0,95	0,95	0,950
Sigma = 8	0,96	0,94	0,95	0,96	0,941
Sigma = 10	0,96	**0,92**	**0,94**	0,95	**0,924**

Bolded values indicate the minimum values achieved for each metric across the different test with noise.

**Table 7:** Test classification performance of noisy and clean images in Fold 1.

Test data	Precision	Recall	F1_score	Accuracy	AUC
Sigma = 0	0,95	0,95	0,95	0,96	0,948
**Sigma = 2**	**0,97**	**0,96**	**0,96**	**0,97**	**0,955**
Sigma = 5	0,95	0,94	0,95	0,95	0,940
Sigma = 8	0,96	0,94	0,94	0,95	0,936
Sigma = 10	0,96	**0,93**	0,94	0,95	**0,928**

Bolded values indicate the minimum values achieved for each metric across the different test with noise.

**Table 8:** Test classification performance of noisy and clean images in Fold 2.

Test data	Precision	Recall	F1_score	Accuracy	AUC
Sigma = 0	0,93	0,94	0,94	0,94	0,941
**Sigma = 2**	**0,95**	**0,95**	**0,95**	**0,96**	**0,948**
Sigma = 5	**0,92**	0,93	0,93	0,94	0,929
Sigma = 8	0,94	0,93	0,93	0,94	0,928
Sigma = 10	0,94	**0,92**	0,93	0,94	**0,924**

Bolded values indicate the minimum values achieved for each metric across the different test with noise.

To assess the impact of class imbalance on the efficiency of the proposed MobileNet_M in this study, we compared the performance of MobileNet_M on imbalanced datasets without data augmentation and after correction. Table 9 shows the comparison. The HEM and ALL accuracy results show that class imbalance affects the generalization of MobileNet_M in favor of the ALL class.

**Table 9:** Test classification performance of MobileNet_M train with imbalance class dataset without data augmentation and MobileNet_M after balancing the dataset.

Metrics	Fold 0		Fold 1		Fold 2	
	MCN	MAC	MCN	MAC	MCN	MAC
ALL precision	0.97	0.97	0.98	0.96	0.96	0.97
HEM precision	0.42	0.95	0.51	0.94	0.37	0.89
Weighted average precision	0.79	0.96	0.83	0.96	0.78	0.94
Accuracy	0.57	0.96	0.69	0.96	0.47	0.94
AUC	0.675	0.954	0.767	0.948	0.609	0.941

### 4.4 Comparison with existing models on ALL

**Figure 8:**
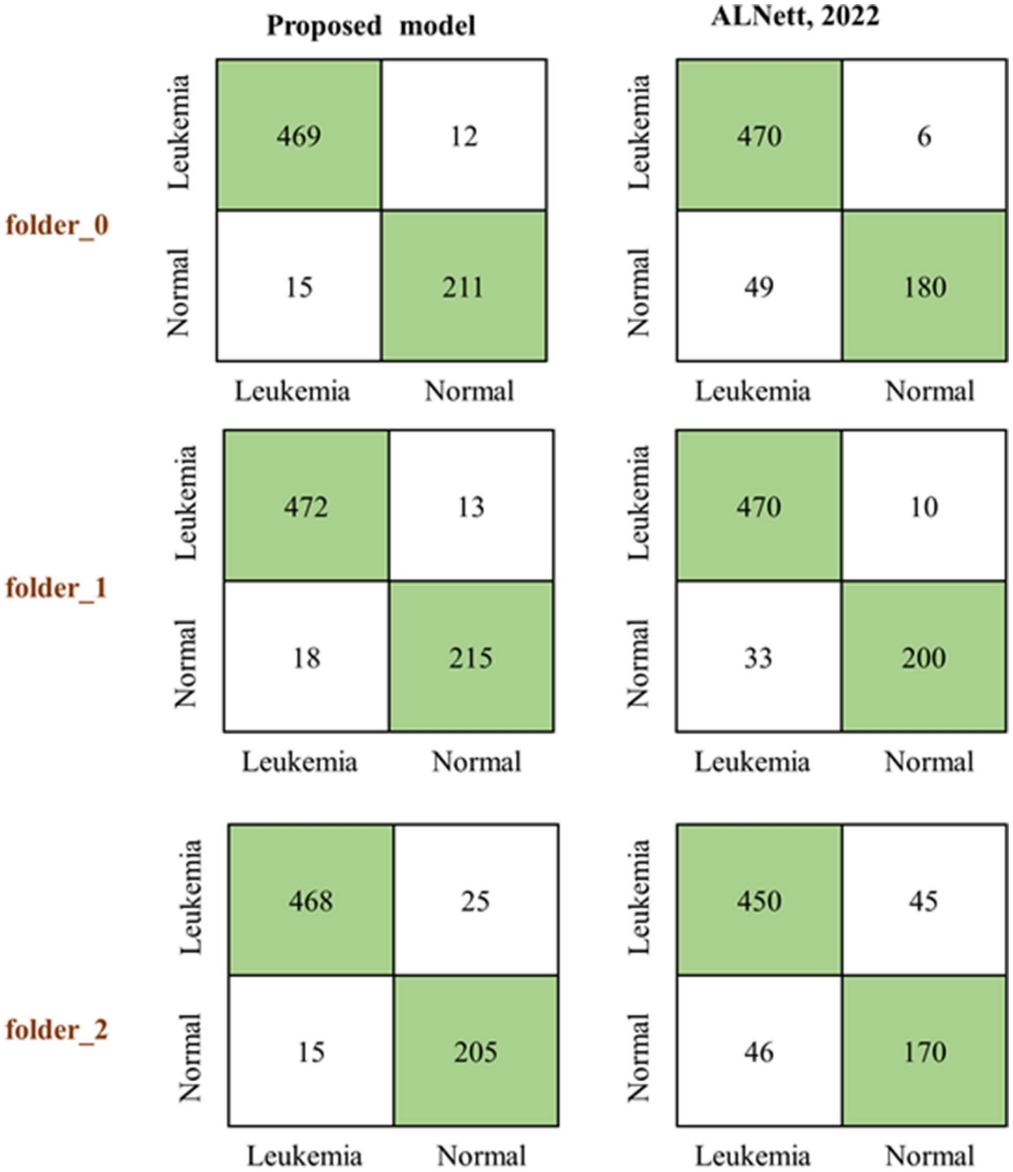
Predicting confusion matrix of the proposed and ALNett models on clean test dataset.

The proposed model was first of all compared to the ALNett model based on confusion matrix and accuracy. The test dataset containing clean images was used for this purpose. ALNett (Jawahar et al., 2022) is a newly established deep convolutional neural network designed for the classification of acute lymphoblastic leukemia. On the given data set, it has shown the highest F1_score and accuracy compared to the ResNet-50, AlexNet, VGG16, and GoogleNet transfer learning models. The accuracy of the developed models compared to ALNett models is shown in Table 10. According to the table, the suggested model’s average accuracy is higher than ALNett’s for each of the three folds. Figure 8 further elucidates this outcome through the confusion matrix, demonstrating that the proposed model surpasses the ALNett model in leukemia detection. For instance, in Fold 0, MobileNet_M identified 680 photos as true positives (TP) and true negatives (TN) with a classification accuracy of 96%, while ALNett classified 650 images as TP and TN with an accuracy of 92.70%.

**Table 10:** Accuracy of prediction of leukemia by the proposed and ALNett.

	Accuracy in %	
	Our model	ALNett, 2022 (Jawahar et al., 2022)
Fold_0	96	92.20
Fold_1	96	94
Fold_2	94	87.20
Average	95.33	91.13

**Figure 9:**
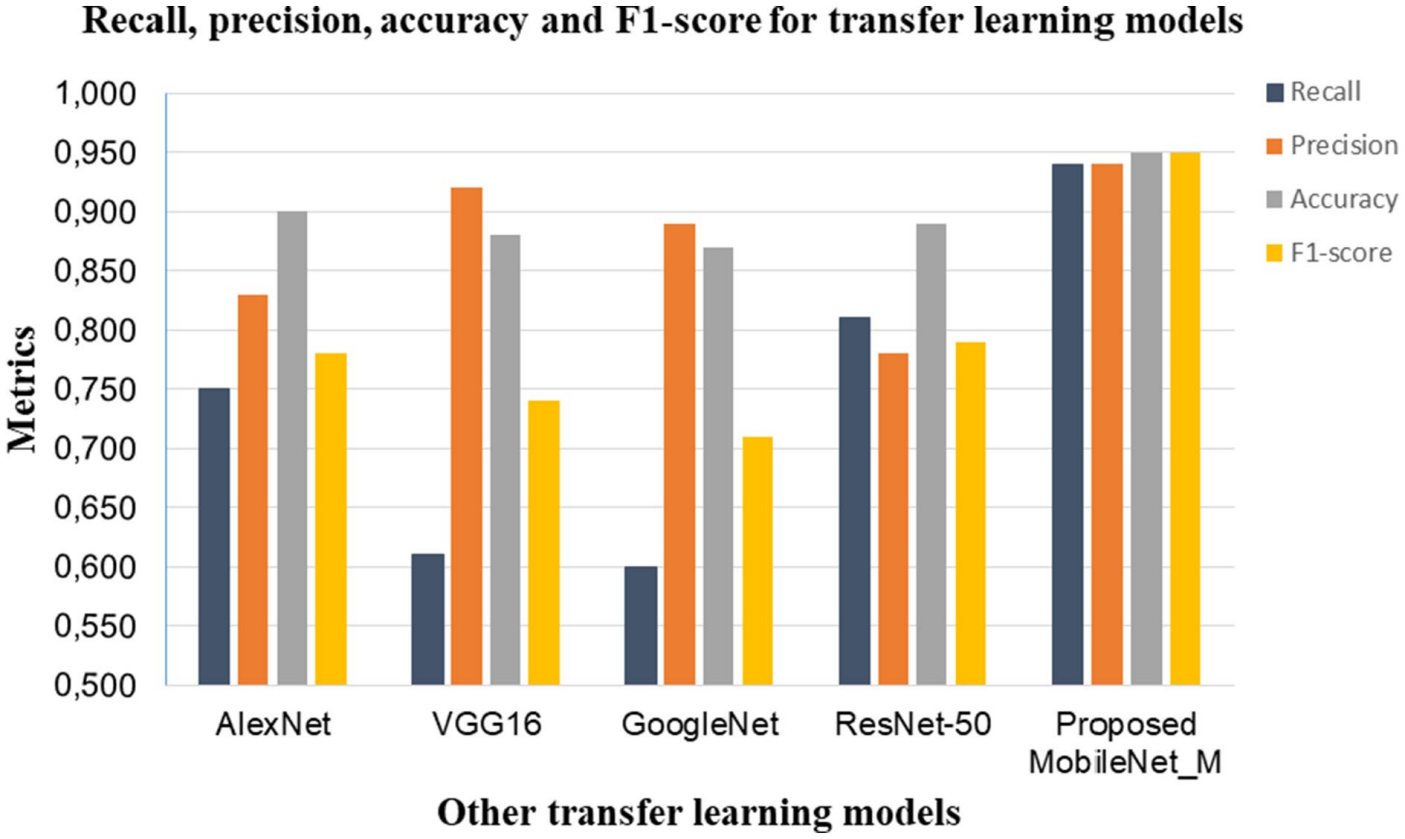
Comparison of common transfer learning models with the proposed MobileNet_M.

Considering the three folds of the dataset, we also compared the average performance (recall, precision, accuracy and F1 score) of the proposed MobileNet_M with transfer learning models such as GoogleNet, ResNet-50, AlexNet and VGG16 reported in Jawahar et al. (2022), as shown in Figure 9. The proposed model obtained the highest recall, precision, accuracy and F1 score values. This comparison reveals the effectiveness of the proposed MobileNet_M.

In other hand, Table 11 presents a comparison of the proposed model with various approaches evaluated on the ISBI 2019 database in recent years. Mathury et al. (2020) achieved 91% of F1_score by overcoming model generalization through the application of local spatial attention blocks learning, pointwise attention convolution layers, and Rademacher Mixup. YOLOv4 was implemented in Khandekar et al. (2021) for cell detection and leukemia classification and achieved F1_score value of 92%. However, as illustrated in Tables 6–8 the proposed MobileNet_M is more robust against additive Gaussian noise achieving better results than the MMA-MTL or YOLO4. This may be due to the proposed balanced database method with specific augmentation process. The results in Tables 10, 11 indicate that the suggested model outperformed contemporary state-of-the-art approaches, achieving an impressive average accuracy of 95.33% and an average F1 score of 95%. The L1 regularization method, the learning rate range, and early stopping callback were key parameters to obtain this performance.

**Table 11:** F1_score comparison of proposed model with existing models.

Authors	Models	Datasets (Rohaziat et al., 2022)	F1_score
Mathury et al. (2020)	MMA-MTL	Training set of C-NMC-NMC -2019	91%
Khandekar et al. (2021)	YOLOv4	Subset of C-NMC-2019	92%
Proposed methods	MobileNet_M	Training set of C-NMC-2019	**95%**

Bold value represents the maximum F1_score.

## 5 Conclusion

This paper proposes a computationally efficient and high-performing model that is resilient to Gaussian noise for the classification of acute lymphoblastic leukemia using microscopic pictures. Thus, the discrimination between these cells is a very challenging task. The pre-trained MobileNet architecture was modified and fine-tuned to address this classification challenge. A new augmentation procedure was proposed both to avoid over-fitting and to build an efficient model. The MobileNet_M model was trained and evaluated using the C_NMC_2019 dataset (Mourya et al., 2019). This study achieved an overall test accuracy of 95.33% and an F1 score of 0.95. The suggested model’s effectiveness and robustness were demonstrated by the introduction of additional Gaussian noise to the test images. The proposed MobileNet_M model yields a better average performance compared to ALNet and several other competitive models.

Based on the results obtained, our suggested model is useful as a guide and second-opinion tool for laboratory technicians and hematologists in the diagnosis of acute lymphoblastic leukemia under a microscope. In the forthcoming period the proposed MobileNet_M model will be deployed on an embedded system or Android phone to build cost-effective devices for computer-assisted diagnosis of leukemia.

## Data Availability

The original contributions presented in the study are included in the article/supplementary material, further inquiries can be directed to the corresponding author.
